# Use of dual-transfection for programmed death cell protein 1 disruption mediated by CRISPR-Cas9 in human peripheral blood mononuclear cells

**DOI:** 10.22038/ijbms.2020.48566.11146

**Published:** 2021-01

**Authors:** SeyedAli Alambeladi, SeyedEbrahim Hosseiny, Mojtaba Jafarinia, Mehdi Dianatpour

**Affiliations:** 1 Department of Genetics, Fars Science and Research Branch, Islamic Azad University, Marvdasht Iran; 2 Department of Genetics, Marvdasht Branch, Islamic Azad University, Marvdasht Iran; 3 Department of Physiology, Shiraz Branch, Islamic Azad University, Shiraz Iran; 4 Department of Medical Genetics, School of Medicine, Shiraz University of Medical Sciences, Shiraz Iran; 5 Stem Cell Technology Research Center, Shiraz University of Medical Sciences, Shiraz Iran

**Keywords:** Cell therapy, Checkpoint protein, CRISPR-Cas9, Induced tolerance, PD-1, PDl-1

## Abstract

**Objective(s):**

Checkpoint blocking is considered a revolutionary method in cancer treatment. This method eliminates cancer cells by maintaining the sensitivity of immune cells. Today, cell therapy through checkpoint blocking is known as the most efficient method of cancer treatment. The programmed cell death protein-1(PD-1), as an immune check protein, has a vital role in weakening the immune responses by reducing the number of stimulated T cells. In normal situations, a decline in the immune responses can cause induced tolerance and prevent autoimmune diseases.

**Materials and Methods:**

In this study, to reduce the induction of tolerance due to PDL-1 binding to PD-1, the PD-1 gene was destroyed in PBMCs by the means of CRISPR-Cas9 and dual-transfection of two plasmids containing the Cas 9 gene and two different sgRNAs specific to two region of PD-1 gene in order to produce a deletion mutation. Six different sgRNA were designed and cloned in PX-458 plasmid vector, and PBMCs were transfected using lipofectamine 2000 and electroporation. Indels were evaluated by gel electrophoresis and Sanger sequencing.

**Results:**

We showed the PD-1 gene in PBMCs was knocked out successfully by CRISPR-Cas9 and dual-transfection of two sgRNAs. The minimum interval between the two sgRNAs was 448 nucleotides.

**Conclusion:**

The results of this research demonstrated that the use of dual-transfection of CRISPR-Cas9 sgRNA is a suitable method to knock out the PD-1 gene and prevention of inducing tolerance in PBMCs.

## Introduction

Cancer is known as one of the most common causes of death worldwide. Checkpoint blocking is considered a revolutionary method in cancer treatment. This method eliminates cancer cells by maintaining the sensitivity of immune cells. Today, cell therapy through checkpoint blocking is known as the most efficient method of cancer control (1, 2). 

The programmed cell death protein-1, also known as PD-1, is the B and T cells surface receptor that binds two ligands named PD-L1 and PD-L2. 

PD-1, as an immune check protein, has a vital role in weakening the immune responses by reducing the number of stimulated T cells. In normal situations, a decline in the immune responses can cause self-induced tolerance and prevent autoimmune diseases (3-5).

Inducing tolerance in T cells that are activated by the binding of PD-L1 to PD-1, happens in two different ways: an increase in apoptosis of activated T cells against cancer cells and a decrease in apoptosis of regulatory T cells (6-9).

In addition to the surface of activated T cells, PD-1 can be found on regulatory T cells. In addition to macrophages and dendritic cells, PD-L1 is also produced by cancer cells in large quantities (1-2). The expression of PD-L2 is more restricted than PD-L1 and this ligand has been proven to be expressed only in dendritic cells and a number of cancer cells. (3-7)

Despite having satisfactory results in cancer treatment, monoclonal antibodies are expensive and difficult to produce; therefore, scientists are forced to search for substitute genetic engineering treatment methods that have more stable results and are much less expensive (4-9).

Peripheral blood mononuclear cells (PBMCs) have a round nucleus. These cells consist of lymphocytes and monocytes. In humans, lymphocytes make up the majority of the PBMCs population. These cells can be extracted from whole blood using ficoll, a hydrophilic polysaccharide that separates layers of blood (10).

The CRISPR-Cas9 tool consists of two parts: the guide RNA and an endonuclease enzyme called Cas9. Guide (sgRNA) is a 20-base long nucleotide sequence that binds to its complementary sequence (target sequence) on DNA. In this study, spCas9 from Streptococcus* pyogenes* (with a PAM sequence of 5’-NGG) was used (6-11).

Su *et al*. (2016) studied the functions of T cells after PD-1 gene inactivation by sending 2 vectors into PBMCs simultaneously (11).

In 2013, llyod *et al*. explained the effects of genome engineering of T cells in cell therapy (12).

In 2015, turning off the PD-1 was introduced as “The best method of immunotherapy for melanoma cancer in the future years” (13-15).

The optimum conditions for gene-delivery for human T cells through electroporation was studied in 2018. (10).

In this study, in order to reduce the inducing tolerance caused by the binding of programmed cell death ligand-1 (PDL-1) to programmed death cell protein-1 (PD-1), we have aimed to knock out the PD-1 gene in PBMCs using CRISPR-Cas9 and simultaneous transfection of two vectors containing two different guide RNA. 

## Materials and Methods

***px458 Vector***

px458 vector (Addgene number 48138) contains a green fluorescent protein gene, ampicillin resistance and a sequence specific for Bbs1 enzyme digestion. The size of this vector is 9288 base pairs. This vector is designed in a way that the guide RNA is located under the U6 promoter. 

***Primers and polynucleotides designing***

Previous studies have shown that simultaneous transfection of two 20 nucleotide sequences for a target gene increases the efficiency of Cas9 performance and reduces the off-target effect (7). 

Considering the probability of attachment inhibition in some parts of the gene, six pair sequences, each containing 20 nucleotides with different distances and a primer pair for flanking regions, were designed and ordered (Table 1).

In order to design the sgRNAs and find the suitable targets, NCBI, CRISPOR and Addgene Guide Designer were used. Moreover, target-inhibited sequences were assessed. sgRNAs which had fewer target-inhibited sequences were selected and ordered.

***Preparation of PBMCs***

10 ml fresh blood in heparin was obtained and using ficoll, PBMCs were separated immediately. By the means of trypan blue staining, the number of living cells per milliliter was calculated and a mixture of 5×10⁵ living cells was added to six-well plates, and sufficient RPMI medium was added instantly. Transfection was performed at 70% confluency of cultured lymphocytes. 

***Lipofectamine***

For transferring the guide RNAs to the PBMCs, the method of “simultaneous entering of two sgRNAs” has been used.

In the plasmid extraction stage, the required vector volume was measured based on optical density. Then, 2.5 μg sgRNA (1.25 μg of each) were calculated for 5 x 10^5^cells in each well and mixed with 10 μg of lipofectamine. Then the mixture of lipofectamine and sgRNAs was slowly added to the PBMCs and incubated at 37 °C and 5% Co_2_. After 5 hr, cells were washed with phosphate-buffered saline and DMEM medium without fetal bovine serum was added.

***Separating transfected cells by fluorescent activated cell sorting (FACS)***

Before extracting the DNA from lymphocytes, transfected cells were separated from the nontransfected cells by a FACS machine (BD FACSAriaᵀᵐ ш). DNA extraction was done immediately and the final concentration was adjusted between 100–200 ng/µl. PCR by Fwd. and Rev. primers was done and the disrupted gene by CRISPR was evaluated by gel electrophoresis on 2% agarose. 

***Electroporation***

The number of PBMCs extracted from the peripheral blood was counted to be about 1× 10⁶, 2.5 µg of each sgRNA, was added to the 2 mm cuvette and the volume was increased to 100 µl by the phosphate-buffered saline. The electroporation machine was set to the single amplitude mode, with a capacitance setting of 1500 µF and a voltage of 220 V. In order to prevent vector enzymatic degradation, an ice-cold container was used throughout all stages. Then, the cells were immediately transferred to a 25 ml flask containing RPMI medium with fetal bovine serum and pen strep. The mixture was incubated at 37 °C in a 5% carbon dioxide atmosphere.

The cells were monitored daily by a fluorescent microscope and were put into the FACS machine after 48 to 72 hr for sorting. The DNA of the separated cells was extracted and subjected to PCR; since the electroporation method is more effective than the lipofectamine, the cleavage band was better in quality.

## Results

Different sgRNAs were designed for different locations within the PD-1 gene, and the sgRNA pairs were entered into PBMCs using two different methods simultaneously (Table 1). The regions of the genome that were expected to be cleaved were amplified by appropriate primers using PCR. Each stage was repeated eight times, and the following results were obtained. 

***Comparison of the simultaneous entrance of sgRNA***

Tables 2 and 3 provide a comparison of the simultaneous entrance of sgRNA3 and sgRNA4 by lipofectamine and electroporation, as well as the length of cleaved fragments and the lack of genomic alteration. As can be observed, there is a significant relationship between the distance of the two guides and the presence of cleavage sites.

***Genome cleavage***

Tables 4 and 5 show genome cleavage by lipofectamine and electroporation, there are two DNA double-stranded breaks after 2,3 and 5 days for sgRNA (3+1) & sgRNA(3+5).

***Effect of vector ʼ s amount***

Tables 6 and 7 present genome cleavage observation in the condition of 3,5 and 5 µg px458 transfected by lipofectamine and electroporation. There is no DNA double-stranded break for sgRNA(3+2), (3+6), (4+1), (4+2), (4+5), (4+6).

***Confirmation of vector entrance:***

**Figure 1:**
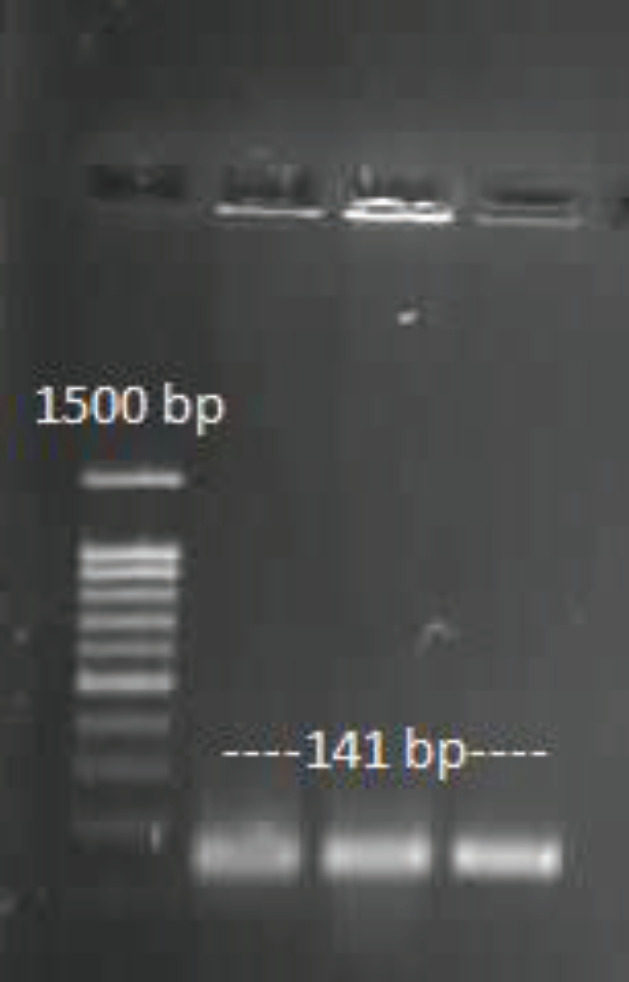
Confirmation of vector entrance into competent cells (colony PCR)

**Figure 2:**
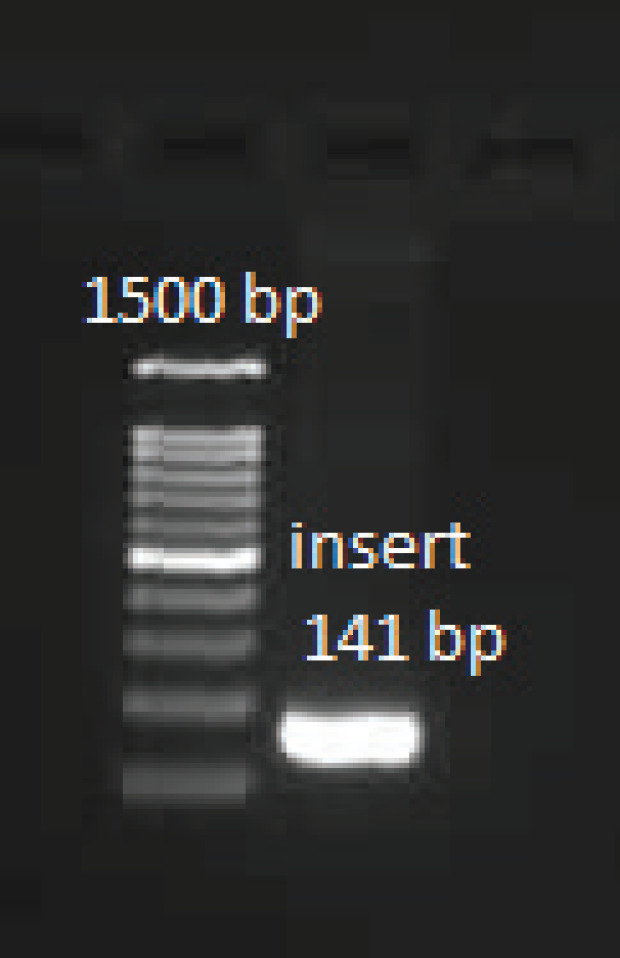
confirmation of insert entrance into the vector

The confirmation of vector entrance into competent cells and insert entrance into vector through colony PCR, are presented in Figures 1 and 2. 

***Entrance of inserts into vectors***

**Figure 3:**
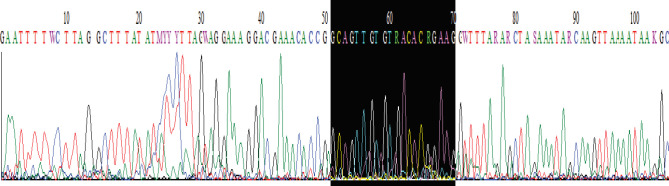
Vector sequencing and insert entrance confirm

Figure 3 demonstrates the process by which the entrance of inserts into vector was confirmed. In both procedures, the vector’s forward primer was utilized, and the insert’s complementary strand was used as the reverse primer.

***Transfection of PBMCs by vector backbone***

**Figure 4:**
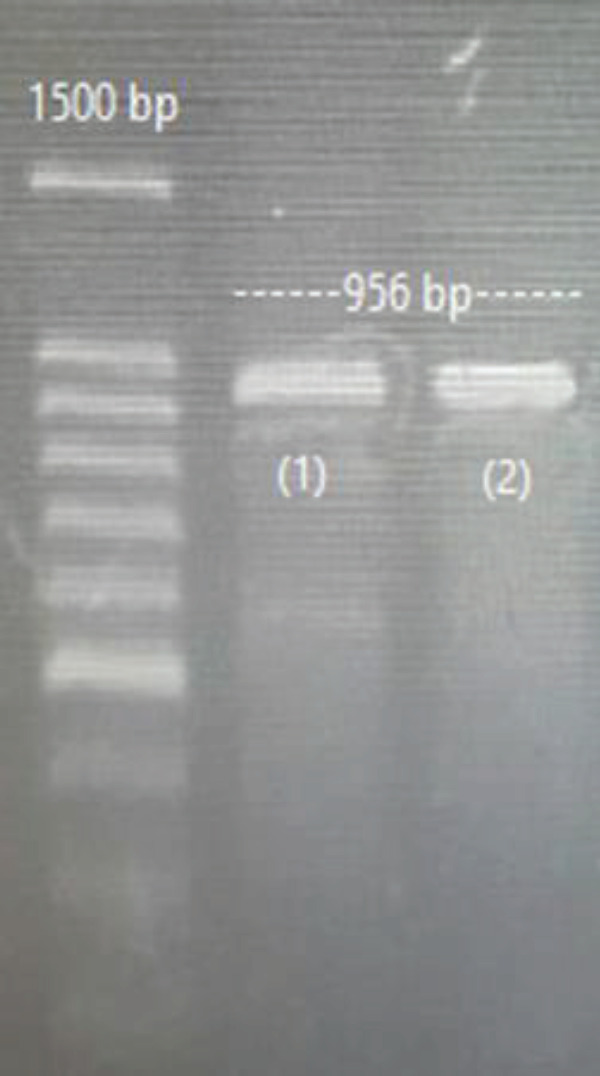
PBMCs transfection by vector backbone (control negative).

Gel electrophoresis of the negative control (transfection of PBMCs by vector backbone), is presented in Figure 4. 

***Observation of cleavage bands***

**Figure 5:**
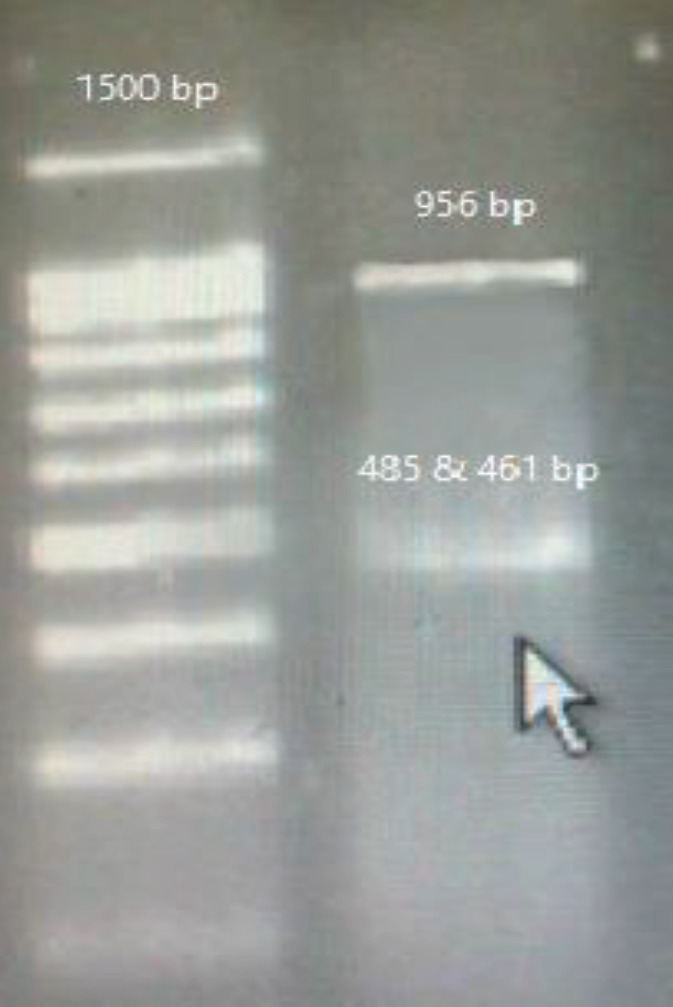
PD-1 knocked out by sg(3+1) and sg(3+5). (lipofectamine)

**Figure 6:**
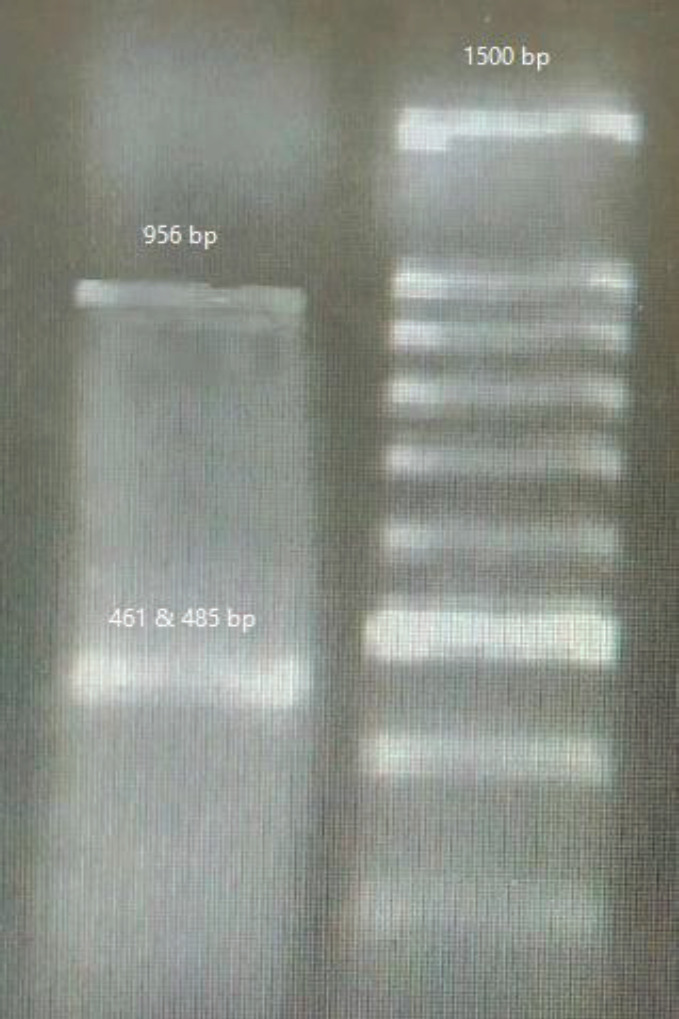
PD-1 knocked out by sg(3+1) and sg(3+5). (electroporation)

Figures 5 and 6 show cleavage bands (PD-1 knockout), sg(3+1), and sg(3+5) were used by lipofectamine and electroporation. Electroporation has more efficiency than lipofectamine, for this reason, the cleavage band is sharper than others. 

***Observation of transfected cells by fluorescence microscope***

**Figure 7:**
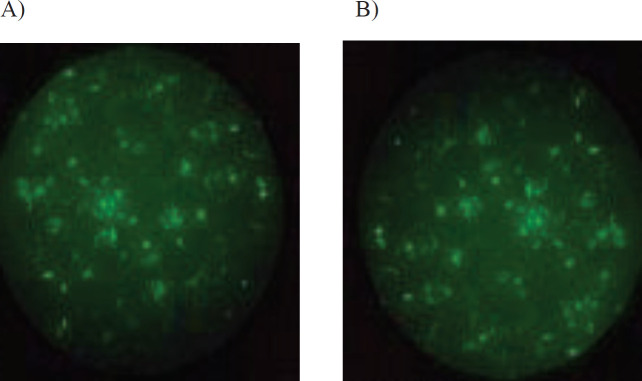
GFP expression was observed 48 hr after transfection by lipofectamine and electroporation

Figure 7 shows that cells had GFP expression after 48 hr in both procedures. 

***Sorting of the transfected cell by FACS machine***

**Figure 8:**
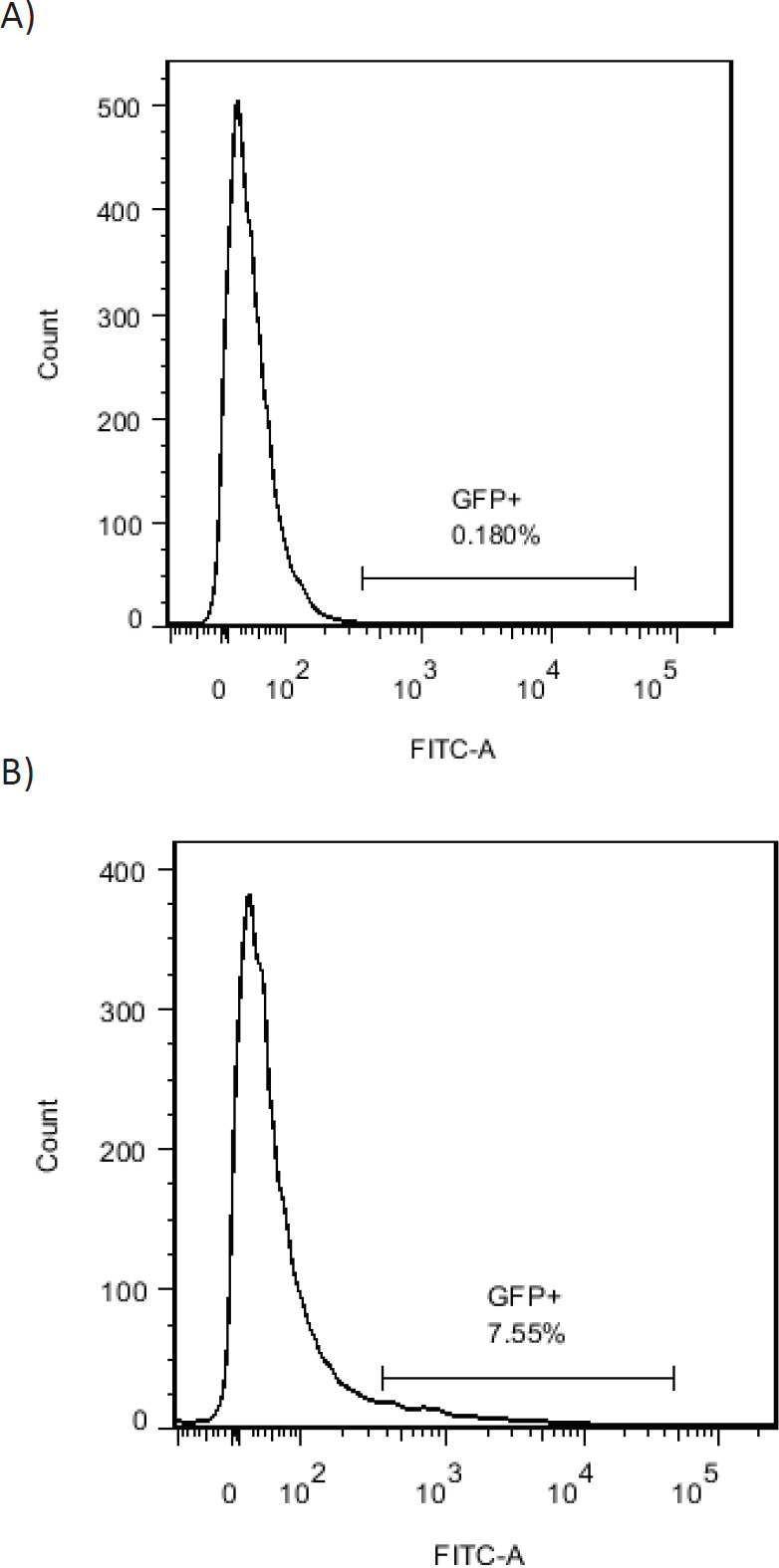
Isolation of transfected PBMCs by FACS. A) Negative control. B) 3-5 days after transfection

Figure 8 shows transfected cell sorting and count by the FACS machine. A) PBMCs without vector. (Negative control). B) cell transfected sorting and count.

***DNA sequencing***

**Figure 9:**
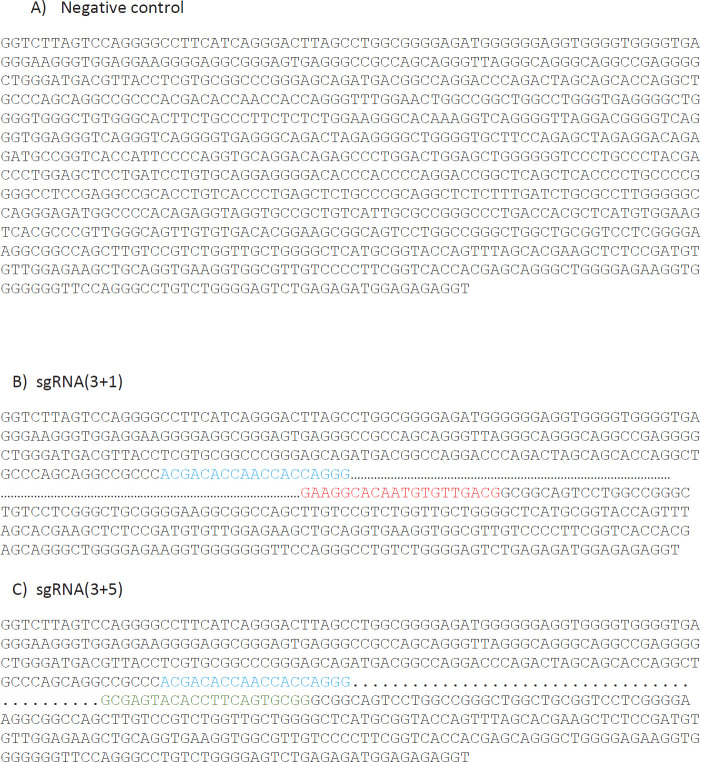
PCR product sequencing: A) Control negative. B) indel produced by sgRNA(3+1) (lipofectamine). C) indel produced by sgRNA(3+5) (electroporation)

Indels are exhibited in Figure 9**: **A) Control negative. B) sgRNA(3+1). C) sgRNA(5+1).

## Discussion

Since the PD-1 gene is expressed naturally in T cells, B cells, and dendritic cells, for its knockout, six different guides were designed and transfected in PBMCs (two guides transfected simultaneously) by applying electroporation and lipofectamine methods (7-11). The px458 vector was chosen so that sgRNA, scaffold, and Cas9 sequences could be transcribed instantaneously. Inspection of PBMCs, after transfection with the px458 vector, using a fluorescent microscope showed that in both electroporation and lipofectamine methods, the vector’s entry into lymphocytes with different guides was satisfactory (Figure 7).

Given that, cleavage bands were observed in simultaneously using sgRNA(1+3) and sgRNA(3+5), it can be said that the distance between the targets is an important factor in cutting the PD1 gene. In other pairs of sgRNAs, we did not observe cleavage bands; this can be the result of the conformational inhibition caused by the two Cas9 proteins reaching close proximity, or due to the presence of heterochromatin areas or epigenetic remodeling (6-18).

In the case of guides 1 and 3, 3 and 5, the appropriate distance between the two guides (487 and 461 nucleotides) prevents the formation of conformational inhibition and therefore, making indels. In the simultaneous use of guides 3 and 2, it is impossible to make an indel, therefore, it seems that the minimum distance between the two sgRNAs must be more than 448 nucleotides for cutting in the PD-1 gene. The difficulty in making a cut in the simultaneous use of other guides can be due to the spatial restraint caused by Cas9 proteins, binding area, or other factors such as heterochromatin regions, epigenetic remodeling, and polymorphism (19-27).

 Guides 3 and 1, 3 and 5, were able to cut the PD-1 gene in both transfection methods, but the other pairs were not. As can be seen in figures 5 and 6, the cleavage band created with the electroporation method is thicker than the lipofectamine method. This could be resulting from electroporation’s higher efficiency in plasmid transfer to PBMCs compare with the lipofectamine method, which has also been mentioned in previous studies (7, 20-31).

In this study, it was shown that by simultaneously entering two guides into the T cell, it is possible to knock out the PD-1 gene and create T cells resistant to induced tolerance resulting from the binding of PDL-1 ligand to the PD-1 receptor.

**Table 1:** Polynucleotides and target locations

sg.No	Polynucleotide	Site of target
1	5´-GCAGTTGTGTAACACGGAAG-3´	241852756 - 241852775
2	5´-GACAGCGGCACCTACCTCTG-3´	241852689 – 241852708
3	5´-ACCCTGGTGGTTGGTGTCGT-3´	241852269 – 241852288
4	5´-TCTCTTTGATCTGCGCCTTG-3´	241852648 – 241852667
5	5´-GGCGTGACTTCCACATGAGCG-3´	241852729 – 241852749
6	5´-GGGCCCTGACCACGCTCATG-3´	241852717 – 241852736
Out-Fwd	5´-GGTCTTAGTCCAGGGGCCTT-3´	241852043 – 241852062
Out-Rev	5´-ACCTCTCTCCATCTCTCAGACT-3´	241852978 – 241852999

Fwd: Forward Primer. Rev: Reverse Primer

**Table 2:** Transfection by lipofectamine

sgRNA	Length of deletion	indel
sg (3+1)	487	+
sg (3+2)	420	-
sg (3+5)	461	+
sg (3+6)	448	-
sg (4+1)	108	-
sg (4+2)	41	-
sg (4+5)	82	-
sg (4+6)	69	-
sg (3+4)	379	-

**Table 3:** Transfection by electroporation

sgRNA	Length of deletion	indel
sg (3+1)	487	+
sg (3+2)	420	-
sg (3+5)	461	+
sg (3+6)	448	-
sg (4+1)	108	-
sg (4+2)	41	-
sg (4+5)	82	-
sg (4+6)	69	-
sg (3+4)	379	-

**Table 4:** Genome cleavage observation

sgRNA	2 days	3 days	5 days
sg (3+1)	+	+	+
sg (3+2)	-	-	-
sg (3+5)	+	+	+
sg (3+6)	-	-	-
sg (4+1)	-	-	-
sg (4+2)	-	-	-
sg (4+5)	-	-	-
sg (4+6)	-	-	-
sg (3+4)	-	-	-

2, 3, and 5 days after transfection by lipofectamine

**Table 5:** Genome cleavage observation

sgRNA	2 days	3 days	5 days
sg (3+1)	+	+	+
sg (3+2)	-	-	-
sg (3+5)	+	+	+
sg (3+6)	-	-	-
sg (4+1)	-	-	-
sg (4+2)	-	-	-
sg (4+5)	-	-	-
sg (4+6)	-	-	-
sg (3+4)	-	-	-

2, 3, and 5 days after transfection by electroporation

**Table 6:** Genome cleavage observation

sgRNA	3 µg	5 µg	7 µg
Sg (3+1)	+	+	+
Sg (3+2)	-	-	-
Sg (3+5)	+	+	+
Sg (3+6)	-	-	-
Sg (4+1)	-	-	-
Sg (4+2)	-	-	-
Sg (4+5)	-	-	-
Sg (4+6)	-	-	-
Sg (3+4)	-	-	-

3, 5, and 7 µg px458 transfected by lipofectamine

**Table 7:** Genome cleavage observation

sgRNA	3 µg	5 µg	7 µg
sg (3+1)	+	+	+
sg (3+2)	-	-	-
sg (3+5)	+	+	+
sg (3+6)	-	-	-
sg (4+1)	-	-	-
sg (4+2)	-	-	-
sg (4+5)	-	-	-
sg (4+6)	-	-	-
sg (3+4)	-	-	-

3, 5, and 7 µg px458 transfected by electroporation

Primers: Fwd. vector̕ s primer. Rev. SgRNAs

Primers: Fwd. vector̕ s primer. Rev. SgRNAs

Fwd. primer: vector̕ s primer

(1): lipofectamine. (2): Electroporation

A: electroporation; B: lipofectamine

## Conclusion

Our study showed that in both electroporation and lipofectamine methods, entry of guide RNAs into the PBMCs is successful. The minimum distance between the two sgRNA must be more than 448 nucleotides for cutting in the PD-1 gene. 

The appropriate distance between the two guides was 487 and 461 nucleotides. In other distances, some factors such as conformational inhibition, heterochromatin remodeling, epigenetics, and polymorphism possibly prevent the PD-1 gene cutting.

Also, the results showed that using the dual-transfection method for PD-1gene knockout in PBMCs, enhances the efficiency of gene cutting and facilitate the detection of cleavage bands.
